# Dysbindin-1 Mutation Alters Prefrontal Cortex Extracellular Glutamate and Dopamine In Vivo

**DOI:** 10.3390/ijms252312732

**Published:** 2024-11-27

**Authors:** Karen K. Szumlinski, Michael C. Datko, Kevin D. Lominac, J. David Jentsch

**Affiliations:** 1 Department of Psychological and Brain Sciences, University of California Santa Barbara, Santa Barbara, CA 93106, USA; mdatko@mgh.harvard.edu (M.C.D.); kevin.lominac@gmail.com (K.D.L.); 2 Department of Molecular, Cellular and Developmental Biology, University of California Santa Barbara, Santa Barbara, CA 93106, USA; 3 Department of Psychology, Binghampton University-State University of New York, Binghampton, NY 13902, USA; jjentsch@binghamton.edu

**Keywords:** dysbindin-1, in vivo microdialysis, prefrontal cortex, extracellular glutamate, extracellular dopamine, NMDA receptors

## Abstract

Elevated risk for schizophrenia is associated with a variation in the *DTNBP1* gene encoding dysbindin-1, which may underpin cognitive impairments in this prevalent neuropsychiatric disorder. The cognitive symptoms of schizophrenia involve anomalies in glutamate and dopamine signaling, particularly within the prefrontal cortex (PFC). Indeed, mice with *Dtnbp1* mutations exhibit spatial and working memory deficits that are associated with deficits in glutamate release and NMDA receptor function as determined by slice electrophysiology. The present study extended the results from ex vivo approaches by examining how the *Dtnbp1* mutation impacts high K+- and NMDA receptor-evoked glutamate release within the PFC using in vivo microdialysis procedures. *Dntbp1* mutant mice are also reported to exhibit blunted K+-evoked dopamine release within the PFC. Thus, we examined also K+- and NMDA-evoked dopamine release within this region. Perfusion of high-concentration K+ or NMDA solutions increased the PFC levels of both dopamine and glutamate in wild-type (WT) but not in *Dtnbp1* mutants (MUT), whereas mice heterozygous for the *Dtnbp1* mutation (HET) exhibited blunted K+-evoked dopamine release. No net-flux microdialysis procedures confirmed elevated basal extracellular content of both glutamate and dopamine within the PFC of HET and MUT mice. These in vivo microdialysis results corroborate prior indications that *Dtnbp1* mutations perturb evoked dopamine and glutamate release within the PFC, provide in vivo evidence for impaired NMDA receptor function within the PFC, and suggest that these neurochemical anomalies may be related to abnormally elevated basal neurotransmitter content.

## 1. Introduction

Schizophrenia is a highly heritable and prevalent neuropsychiatric disorder characterized by severe impairments in cognition that limit psychosocial function [1,2]. Many promising candidate genes have been identified for schizophrenia susceptibility [3,4], of which the gene encoding dystrobrevin-binding protein-1 (dysbindin-1 or *DTNBP1*) [5,6] has received considerable experimental attention. *DTNBP1* lies within the chromosome 6p24-22 susceptibility locus [7,8]. Schizophrenia has been associated with several single nucleotide polymorphisms or haplotypes of *DTNBP1* [3,6,9] and analyses based on gene-wide association studies, coupled with other genetic and gene expression studies in humans and animals, highlighted *DTNBP1* as a susceptibility gene for schizophrenia [3]. In particular, *DTNBP1* risk polymorphisms are more common in individuals with earlier adult-onset schizophrenia, characterized by pronounced cognitive deficits and clinical symptoms [10]. Further, abnormal *DTNBP1* gene expression is reported within the dorsolateral prefrontal cortex (dlPFC) and hippocampus in schizophrenia [11,12,13,14,15], and dysbindin-1 protein expression is reported consistently as reduced within both structures [11,12,13]. Finally, sandy (Sdy) mice that carry an in-frame 22-residue deletion wholly within the *Dtnbp1* gene [16] exhibit a host of behavioral anomalies, including deficits in social interactions, impaired habituation, enhanced stimulant sensitization and impaired cognitive performance in fear-conditioning, novel object recognition, spatial reference, and working memory [17,18,19,20,21,22], that are consistent with a model of schizophrenia-relevant cognitive and behavioral impairment. To complement these studies, we demonstrated deficits in spatial working memory function, an important endophenotype for schizophrenia [1,2,23].

Dysbindin-1 is part of the Biogenesis of Lysosome-related Organelle Complex 1 (BLOC-1 complex) [24], which regulates synaptic vesicle dynamics through stabilization of the t-SNARE complex [19,25,26,27,28,29]. Further, dysbindin-1 regulates the expression of several proteins key to calcium-dependent vesicle mobilization to the active zone and vesicle priming, including snapin, synapsin 1, syntaxin I and II, synaptotagmin-1, and L- and N-type calcium channels [29,30]. Dysbindin-1 is expressed in dopaminergic and glutamatergic neurons in the brain and has been localized to both pre- and postsynaptic elements of glutamatergic cells [6,12,31]. In vitro, dysbindin-1 knockdown alters extracellular dopamine and glutamate levels in PC12 cells [30,32]. Corresponding to their abnormal behavioral phenotype, Sdy mice or *Dtnbp1* mutant mice on a C57BL/6 background are reported to have lower tissue dopamine content [20,33], higher extracellular dopamine content [34,35], and blunted high potassium (K+)-evoked dopamine release within the PFC when assayed using in vivo microdialysis procedures [36]. Sdy mice also exhibit many indices of impaired glutamate release in ex vivo slice preparations from PFC, including reduced paired-pulse facilitation, reduced evoked and miniature excitatory postsynaptic potentials, a smaller ready releasable pool, smaller quantal size, a lower probability of release and a slower recovery of the ready-releasable pool [22,29], as well as lower expression and function of the NMDA-type glutamate receptors [37]. How the null dysbindin mutation impacts glutamate release in vivo is yet unknown, nor is it known whether dysbindin mutation alters in vivo basal extracellular dopamine and glutamate content within the PFC.

Using in vivo microdialysis procedures, we detected elevated extracellular levels of both glutamate and dopamine within the medial PFC (mPFC) of mice heterozygous (HET) or homozygous (MUT) for the null dysbindin-1 mutation, and these results were confirmed using no net-flux procedures. Further, we show that neither high K+ nor NMDA perfusion evoked glutamate or dopamine release within the mPFC of MUT mice, whereas HET mice exhibited impaired dopamine release only, demonstrating haploinsufficiency for these effects. The present results corroborate prior indications that *Dtnbp1* mutations perturb evoked dopamine and glutamate release within the PFC, provide in vivo evidence for impaired NMDA receptor function within the mPFC, and suggest that these neurochemical changes may be related to abnormally elevated basal neurotransmitter content.

## 2. Results

### 2.1. Depolarization-Induced Neurotransmitter Release

**Figure 1:**
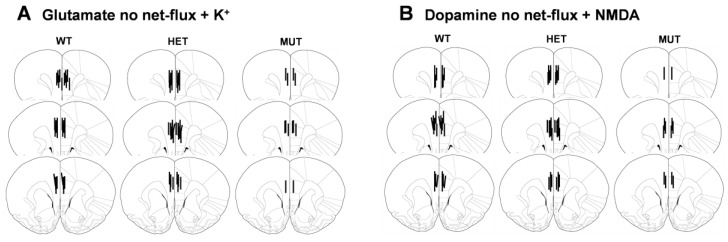
Cartoon of the location of the active membranes of the microdialysis probes of wild-type (WT), heterozygous mutant (HET), and homozygous mutant (MUT) mice in the study of (**A**) K+-evoked neurotransmitter release and glutamate no net-flux and (**B**) NMDA-evoked neurotransmitter release and dopamine no net-flux.

We first extended the results of ex vivo glutamate release [22,29] and in vivo dopamine release studies [34,35] by comparing the capacity of high K+ solutions (50 and 100 mM), perfused via the microdialysis probe, to depolarize neurons and elicit dopamine and glutamate release within the PFC of *Dtnbp1* WT, HET, and MUT mice on a C57BL/6J background. As depicted in Figure 1A, the active membrane of the microdialysis probes was localized to the mPFC, with no overt differences between WT, HET, and MUT mice that underwent drug infusion procedures.

**Figure 2:**
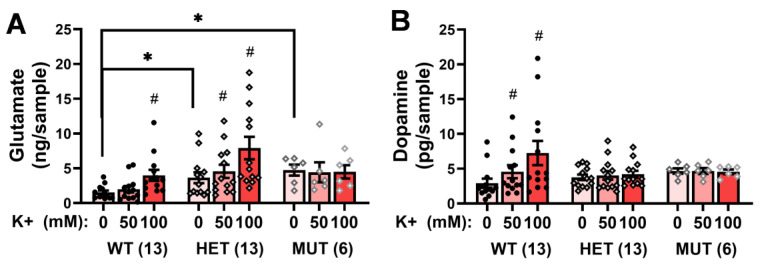
Comparison of the effects of perfusion of K+ solutions (0, 50, and 100 mM) into the mPFC of wild-type (WT), heterozygous mutant (HET), and homozygous mutant (MUT) mice on (**A**) extracellular levels of glutamate and (**B**) extracellular levels of dopamine. The data represent the means +/− SEMs of the number of mice indicated in parentheses. * *p* < 0.05 vs. WT (Genotype effect; SNK post-hoc tests); # *p* < 0.05 vs. 0 mM (K+ effect; corrected *t*-tests).

As prior studies indicated altered basal dopamine content in both Sdy mice [20,33,34] and *Dtnbp1* mutant mice on a C57BL/6 background [35], we conducted a priori comparisons of baseline glutamate and dopamine levels (i.e., 0 mM K+). Both HET and MUT mice exhibited significantly elevated baseline glutamate relative to WT controls (Figure 2A) [0 mM: Genotype effect: F(2,31) = 7.06, *p* = 0.003; SNK post-hoc tests]. Although it appeared that the *Dtnbp1* mutation also had elevated baseline dopamine levels (Figure 2B), no significant group differences were detected at the 0 mM K = concentration (univariate ANOVA, *p* = 0.16). These data suggested that a *Dtnbp1* mutation regulates basal extracellular glutamate levels within the mPFC, with a weaker effect on extracellular dopamine.

The capacity of the local perfusion of the mPFC with high K+ solutions to elevate extracellular levels of both glutamate (Figure 2A) and dopamine (Figure 2B) depended upon genotype as indicated by significant Genotype X Concentration interactions [for glutamate, F(4,58) = 3.09, *p* = 0.02; for dopamine, F(4,54) = 3.67, *p* = 0.01]. As illustrated in Figure 2A, the 100 mM K+ solution approximately doubled the mPFC glutamate levels in both WT [F(2,24) = 20.30, *p* < 0.0001; 0 vs. 50 mM: *p* = 0 vs. 100 mM: *p* < 0.001] and HET mice [F(2,24) = 7.32, *p* = 0.003; 0 vs. 50 mM: *p* = 0.09; 0 vs. 100 mM: *p* = 0.006]. In contrast, depolarization-induced glutamate release was completely absent in MUT animals (for both concentrations *p* ≥ 0.80).

An examination of K+-stimulated dopamine release also indicated genotypic differences (Figure 2B) [Genotype X Concentration: F(4,58) = 5.108, *p* = 0.001]. As observed for glutamate, the 100 mM K+ solution approximately doubled the mPFC dopamine levels in WT mice (Figure 2B) [F(2,24) = 8.63, *p* = 0.002; 0 vs. 50 mM: *p* = 0.046; 0 vs. 100 mM: *p* = 0.009], and this effect was also absent in MUT animals (*p* = 0.90). Interestingly, despite exhibiting a WT-like glutamate response to high K+, HET animals failed to exhibit depolarization-induced increases in mPFC dopamine (*p* = 0.25). These data obtained using in vivo microdialysis approaches corroborate and extend those obtained from in vitro and ex vivo studies [22,29,35] by demonstrating that dysbindin-1 is critical for both dopamine and glutamate neurons within the mPFC in vivo.

### 2.2. NMDA-Induced Neurotransmitter Release

**Figure 3:**
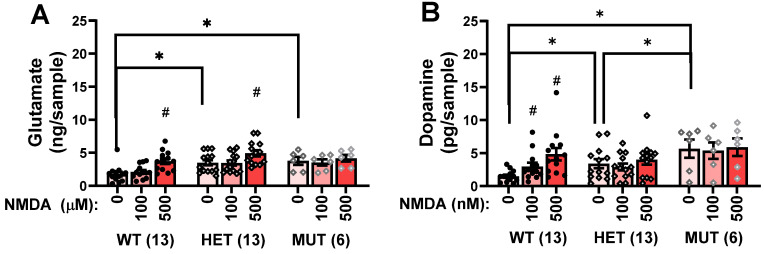
Comparison of the effects of perfusion of NMDA-containing solutions (0, 100, and 500 μM) into the mPFC of wild-type (WT), heterozygous mutant (HET), and homozygous mutant (MUT) mice on (**A**) extracellular levels of glutamate and (**B**) extracellular levels of dopamine. The data represent the means +/− SEMs of the number of mice indicated in parentheses. * *p* < 0.05 vs. WT (Genotype effect; SNK post-hoc tests); # *p* < 0.05 vs. 0 mM (NMDA effect; corrected *t*-tests).

A dysbindin-1 mutation disrupts NMDA-evoked currents in slice preparations from the mPFC and reduces the mRNA expression of the obligatory GluN1 subunit of the NMDA receptor [37]. Thus, we also determined whether MUT mice exhibited deficits in NMDA receptor function in vivo by examining for NMDA-evoked (100 and 500 μM) glutamate and dopamine release within the mPFC. Again, we detected elevated baseline glutamate levels within the mPFC of HET and MUT mice, compared to WT controls (Figure 3A; 0 μM) [F(2,31) = 10.55, *p* < 0.0001; post-hoc tests]. However, in this experiment, the *Dtnbp1* mutation increased baseline dopamine in a gene dose-dependent manner (Figure 3B; 0 μM) [F(2,31) = 8.614, *p* = 0.001; post-hoc tests: WT < HET < MUT].

Intra-mPFC perfusion of NMDA elicited a rise in extracellular glutamate levels that also depended on genotype [F(4,58) = 4.12, *p* = 0.005]. As illustrated in Figure 3A, 500 µM of NMDA elevated significantly glutamate levels in both WT [F(2,24) = 32.91, *p* < 0.0001; 0 vs. 100 µM: *p* = 0.06; 0 vs. 500 µM: *p* < 0.001] and HET animals [F(2,24) = 20.61, *p* < 0.0001; 0 vs. 500 µM: *p* < 0.001], but neither NMDA concentration affected glutamate levels in MUT mice (*p* = 0.07). The *Dtnbp1* mutation also altered NMDA-stimulated dopamine release in the mPFC [Genotype X NMDA: F(4,58) = 4.14, *p* = 0.005]. As illustrated in Figure 3B, intra-mPFC NMDA dose-dependently elevated dopamine levels in WT mice [F(2,24) = 10.50, *p* = 0.001; 0 vs. 100 µM: *p* = 0.002; 0 vs. 500 µM: *p* = 0.002], whereas no significant NMDA effect was apparent in either HET (*p* = 0.12) or MUT (*p* = 0.44) animals. Together, these in vivo microdialysis data for NMDA-evoked glutamate release indicate that the *Dtnbp1* mutation blunts receptor function in a freely moving animal, extending prior ex vivo evidence for impaired NMDA receptor function within the PFC [36].

### 2.3. No Net-Flux Microdialysis

The above results obtained using conventional microdialysis procedures suggested that the *dtnbp1* mutation elevated basal extracellular levels of glutamate and dopamine within the mPFC. As the results from conventional microdialysis procedures are subject to differences in individual probe recovery, we conducted no net-flux microdialysis studies to confirm genotypic differences in neurotransmitter content independent of probe recovery [38].

**Figure 4:**
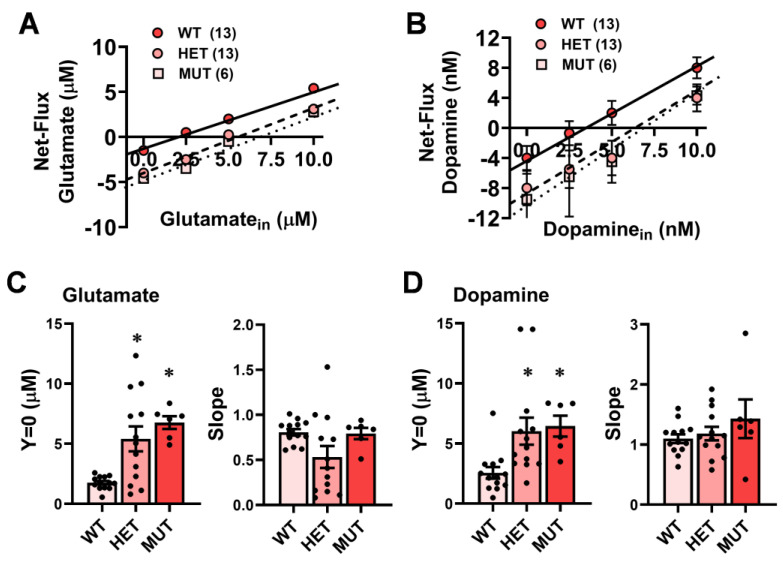
Summary of the results of the no net-flux microdialysis experiments in which (**A**) glutamate and (**B**) dopamine were perfused into the mPFC of wild-type (WT), heterozygous mutant (HET) and homozygous mutant (MUT) mice. (**C**) Comparison of the x-intercept (y = 0; estimate of basal neurotransmitter content) for glutamate (left) and the slopes of the linear regression for the glutamate study (reflection of clearance/reuptake; right) between WT, HET, and MUT mice. The data in panel C is derived from panel A. (**D**) Comparison of y = 0 (left) and the slopes of the linear regressions (right) from the dopamine study. The data in panel D are derived from panel B. The data represent the means +/− SEMs of the number of mice indicated in parentheses. * *p* < 0.05 vs. WT (Genotype effect; SNK post-hoc tests).

The results of our no net-flux experiments are depicted in Figure 4A for glutamate and Figure 4B for dopamine. Confirming earlier observations, marked genotypic differences were observed for the estimate of the basal extracellular content of both neurotransmitters as derived from the x-intercepts (y = 0) of linear regression analyses conducted on the plots of the net-flux of both glutamate (Figure 4C) and dopamine (Figure 4D) versus the neurotransmitter perfused through the probe [for glutamate: F(2,30) = 5.81, *p* = 0.008; for dopamine: F(2,29) = 4.78, *p* = 0.02]. As depicted in Figure 4C and Figure 4D, respectively, the basal extracellular levels of both glutamate and dopamine were approximately double in HET and MUT mice compared to WT controls, and post-hoc analyses confirmed these group differences. However, group differences were not observed regarding the slopes of the linear regressions for either glutamate (Figure 4C) or dopamine (Figure 4D) (univariate ANOVAs, *p*’s > 0.3 for both neurotransmitters), indicative of no effect of the *Dtnbp1* mutation on glutamate release or reuptake mechanisms [38]. Thus, the *Dtnbp1* mutation elevates basal extracellular content of both glutamate and dopamine within the mPFC without overtly impacting mechanisms of clearance or release, at least as determined using this microdialysis procedure.

## 3. Discussion

Here, we provide the first in vivo evidence that constitutive *Dtnbp1* deletion elevates extracellular glutamate and dopamine content concomitantly within the mPFC. We also replicate in mice on a C57BL/6J background the blunted K+-evoked dopamine release within the mPFC reported for dysbindin-1 mutants on a DBA/2J background [36] and extend these findings to mPFC glutamate. Finally, we provide in vivo validation of impaired NMDA receptor function within the mPFC of dysbindin-1 mutants [37,39] as evidenced by blunted NMDA-evoked release of both glutamate and dopamine within this region.

As a member of the BLOC-1 complex [24], dysbindin-1 is theorized to impair glutamate release via the regulation of synaptic vesicle dynamics and the stabilization of the t-SNARE complex [25,26,27]. The impaired K+- and NMDA-evoked glutamate release within the mPFC of MUT mice observed herein (Figure 2A and Figure 3A) align with prior in situ studies of dysbindin-1 mutants indicating impaired glutamate release [22,29,30,40]. In these prior studies, impaired evoked glutamate release in dysbindin-1 mutants has been related to a smaller readily releasable pool of glutamate, lower probability of release, and slower recovery of the readily releasable pool [29,40]. While the precise molecular mechanisms contributing to abnormal mPFC glutamate release in dysbindin-1 mutants are not fully understood, the trafficking of synaptic vesicles from the reserve to the readily releasable pool requires calcium influx and accumulation. Null dysbindin-1 mutants exhibit an approximately 30% reduction in depolarization-evoked calcium influx in synaptosomal preparations from the PFC, as well as lower expression levels of both N- and L-type calcium channels relative to WT mice [29]. In addition to this primary deficit in calcium entry, null dysbindin-1 mutation lowers the expression of another component of the BLOC-1 complex, snapin [29,40], which normally stabilizes SNAP25 interactions with the calcium-sensitive vesicular protein synaptotagmin-1 to prime vesicles for release [30]. Although dysbindin-1 mutation does not appear to affect SNAP 25 levels within the mPFC [29], immunoblotting conducted on synaptosomal fractions from the PFC of null dysbindin-1 mutants revealed gross perturbations in the expression levels of many key proteins involved in the calcium-dependent mobilization and docking of synaptic vesicles, including the adaptor protein AP3, both the non-phosphorylated and phosphorylated forms of syntaxin 1, synaptotagmin-1, as well as both synapsin I and II [29]. Such anomalies in the expression of proteins critical for vesicular glutamate release can readily account for impaired glutamate transmission within the PFC associated with the behavioral anomalies expressed by Sdy or other dysbindin-1 mutants, e.g., [17,18,19,20,21,22].

However, impairments in vesicular trafficking do not readily account for the higher basal extracellular glutamate content observed within the mPFC of HET and MUT mice in this study relative to WT controls (Figure 4A,C). To the best of our knowledge, this is the first demonstration of *Dntpb1* haploinsufficiency with respect to maintaining basal extracellular glutamate content within any brain region, and it will be important in future work to extend these results to other brain regions. Basal extracellular glutamate content is regulated, in part, by the metabotropic mGlu2/3 autoreceptors [41], a dysfunction that has been implicated in the neuropathology of schizophrenia, e.g., [42]. It is worth noting that despite the higher basal extracellular glutamate exhibited by both HET and MUT mice, only MUT mice failed to exhibit evoked glutamate release at the highest concentration of both K+ (Figure 2A) and NMDA (Figure 3A). Thus, the effects of *Dntbp1* deletion on evoked glutamate release are dissociated from basal extracellular glutamate tone on autoreceptors. Basal extracellular glutamate content is also regulated by sodium-dependent and -independent transporters, e.g., [43,44], which are theorized to be druggable targets for antipsychotic therapy, e.g., [42,43,44,45]. While *Dntbp1* mutation is reported to blunt mGlu1/5 function [46], we are unaware of any study examining the role of dysbindin-1 in regulating the function of mGlu2/3 autoreceptors or glutamate transporters. However, dysbindin-1 is expressed by astrocytes [47], which contribute majorly to the regulation of extracellular glutamate in the brain [48]. Thus, it is entirely possible that an imbalance in glial-mediated glutamate influx/efflux might contribute to the heightened glutamate levels observed herein.

Although the molecular mechanisms through which dysbindin-1 mutation impacts synaptic vesicle dynamics, docking, and neurotransmitter release have been characterized primarily within the context of glutamate transmission in the PFC and hippocampus [22,29,30,40], such deficits in calcium influx, coupled with anomalies in the expression of many proteins involved in synaptic vesicle mobilization and priming are not specific to glutamatergic neurons. Indeed, the present data (Figure 2B and Figure 3B), coupled with the results of a prior in vivo microdialysis study of Sdy mice [36], indicate a clear deficit also in K+-evoked dopamine release within the PFC. One additional contributing mechanism to blunted evoked dopamine release within the PFC exhibited by null dysbindin-1 mutants may reflect changes in the cell surface expression and signaling of D2-type dopamine receptors, the major autoreceptor regulating dopamine release throughout the brain c.f., [49]. Dysbindin-1 reduces the cell surface expression of D2 receptors, in addition to lowering the potency of dopamine to recruit its major intracellular effector, adenylyl cyclase, and to induce the phosphorylation of downstream effectors (e.g., ERK1/2 and Akt) [50]. Conversely, Sdy mice exhibit elevated cell surface expression of D2 receptors within the PFC [51], and siRNA-mediated *Dntbp1* knock-down prevents dopamine-induced D2 receptor internalization in cell culture systems that normally contribute to autoreceptor desensitization [50]. Herein, both *Dntpb1* HET and MUT mice exhibited higher basal extracellular dopamine content within the PFC than WT controls (Figure 4B,D), a finding consistent with prior studies of baseline dopamine levels in dysbindin-1 mutants [20,33,34,35]. Although we did not assay directly for changes in the PFC D2 autoreceptor function herein, one can readily envision how a failure of autoreceptor desensitization [50], particularly in the face of consistently high dopamine tone (Figure 4B,D) [34,35], would maintain inhibition on evoked dopamine release in this region. Indeed, the capacity of the D2/D3 receptor agonist quinpirole to reduce extracellular dopamine within the PFC is intact in heterozygous dysbindin-1 mutants despite higher baseline dopamine levels [34].

However, neither reduced sensitivity to agonist-induced autoreceptor desensitization nor disrupted vesicular trafficking/docking/priming can readily account for the elevated basal extracellular dopamine content observed within the PFC of either HET and MUT mice (Figure 4B,D) and reported previously [34]. Given dysbindin-1′s purported role in the trafficking, internalization, and recycling of proteins at the level of the cell membrane, e.g., [50], it is possible that dysbindin-1 mutation lowers the cell surface expression of plasmalemmal transporters (e.g., DAT and NET) that are responsible for clearing dopamine from the synaptic cleft, e.g., [52]. Indeed, dysbindin-1 mutation impairs amphetamine-induced dopamine release within both the mPFC [53] and the nucleus accumbens [54], which is impulse-independent and requires intact DAT/NET function [55,56]. However, conditional deletion of dysbindin-1 from dopamine neurons does not alter cytosolic or plasma membrane DAT expression, at least within the nucleus accumbens [57]. How dysbindin-1 mutation impacts DAT or NET expression within the mPFC has not been described to the best of our knowledge, although Sdy mice exhibit blunted norepinephrine release from adrenal chromaffin cells [40]. Given that NET is the major mechanism controlling dopamine reuptake in the PFC and, therefore, critical for regulating basal dopamine content [58], an important step in future work should be a more thorough characterization of how the dysbindin-1 mutation influences noradrenergic transmission within the PFC of relevance to its role in the cognitive symptomology of schizophrenia [59,60]. Alternatively, dysbindin-1 mutation might influence the function/activity of catechol-*O*-methyltransferase (COMT), an enzyme that is critical for catecholamine degradation, particularly within the PFC [61]. While there is evidence from both mice and humans for a non-linear epistatic interaction between the genes encoding dysbindin-1 and COMT [54,62], there is no direct evidence of which we are aware indicating a direct effect of dysbindin-1 mutations on COMT function to account for the elevated basal extracellular dopamine content of heterozygous and homozygous *Dntbp1* mutant mice (Figure 4B,D) [34,35].

Constitutive *Dtnbp1* mutants exhibit disrupted NMDA-evoked currents in slice preparations from the mPFC, in addition to reduced mRNA expression of the obligatory GluN1 subunit of the NMDA receptor [37]. More recently, a study of mice with a conditional deletion of *Dtnbp1* within presumably glutamatergic CaMKIIα-positive cells revealed impaired spatial learning/memory, coupled with a blunted capacity of the NMDA receptor antagonist MK801 to induce locomotor activity and to impair prepulse inhibition of acoustic startle, indicative of NMDA receptor dysfunction [39]. Consistent with this, these CaMKIIα conditional knock-out mice exhibit reduced protein expression of GluN1 and GluN2b receptor subunits within both the PFC and hippocampus [39]. While we did not examine protein expression of NMDA receptor subunits herein, our observations of blunted NMDA-evoked dopamine and glutamate release within the mPFC provide in vivo functional evidence for impaired NMDA receptor function in this region and, importantly, indicate that this impairment impacts both glutamate and dopamine release. In the mPFC, NMDA receptors are located primarily on the dendrites of pyramidal neurons [56,63], but there is evidence supporting the presynaptic localization of NMDA receptors on dopamine terminals in the PFC [64] and on glutamatergic terminals in other neocortical regions (e.g., entorhinal cortex, visual cortex, amygdala and hippocampus) [65], with receptor stimulation inducing increased indices of neurotransmitter release. Whether the blunted NMDA-evoked dopamine and glutamate release reflect a dysbindin-1 effect on the expression of presynaptically localized NMDA receptors is a viable hypothesis to account for the simultaneous dysregulation of both neurotransmitters. Evidence also indicates a functional interaction between D2 and NMDA receptors by which the activation of the D2 receptor can inhibit NMDA receptor function [66]. Thus, if dysbindin-1 mutation augments D2 receptor expression/function as discussed above, this might apply to its capacity to impair NMDA receptor function above and beyond effects on the trafficking of the NMDA receptor per se.

The present in vivo microdialysis extends the current literature regarding the role of dysbindin-1 in dopamine and glutamate neurotransmission by demonstrating the concurrent dysregulation of both dopamine and glutamate within the mPFC of *Dntbp1* mutant mice on a C57BL/6J genetic background. This dysregulation is apparent with respect to the basal extracellular content of these neurotransmitters and their release evoked by depolarization or NMDA receptor stimulation. While it remains to be determined if and precisely how anomalies in mPFC glutamate relate to anomalies in dopamine, these findings augment the construct validity of the *Dntbp1* knock-out mouse as a partial animal model of schizophrenia with which to study the underlying cellular and molecular pathology of this highly prevalent neuropsychiatric disorder.

## 4. Materials and Methods

### 4.1. Subjects

Studies were male and female mice carrying a large genomic deletion (exons 6–7; introns 5–7 within the *Dtnpb1* gene [16] that were backcrossed to the C57Bl/6J background (Jackson Laboratory, Bar Harbor, ME, USA). Mice were genotyped as previously described [22], and WT, HET, and MUT littermates, aged approximately 45–60 days, were employed in the current experiments. All experimental protocols were approved by the Chancellor’s Animal Research Committee at the University of California Los Angeles.

### 4.2. Surgery

The surgical procedures to implant bilateral guide cannulae above the mPFC were identical to those employed by our group, e.g., [67]. Under isoflurane anesthesia, guide cannulae (20-gauge, 7 mm long; Small Parts, Roanoke, VA, USA) were implanted 2 mm over the mPFC using the following coordinates from the mouse brain atlas of Paxinos and Franklin (2004) [68]: AP: +2.0 mm; ML: ±0.5 mm; DV: −1.0 mm from Bregma and affixed to the skull using dental resin. To prevent continuous externalization, dummy cannulae (24 gauge; length equivalent to guide cannulae) were placed inside the guide cannulae and only removed before in vivo microdialysis procedures (see below). Following the completion of a microdialysis session, the dummy cannulae were immediately reinserted. Animals were allowed a minimum of 7 days of recovery from surgery prior to commencing experimental procedures.

### 4.3. In Vivo Microdialysis

In vivo microdialysis was conducted to assess the impact of gene deletion on the sensitivity of dopamine and glutamate terminals within the mPFC to the local perfusion of depolarizing, high K+ solutions (0–100 mM) and stimulation of NMDA receptors using NMDA (0–100 nM). We also determined the effect of gene deletion on the basal extracellular content of dopamine and glutamate within the mPFC using no net-flux procedures (2.5, 5, and 10 μM of glutamate or 2.5, 5, and 10 pM of dopamine), akin to those described previously, e.g., [67,69,70]. For all experiments, microdialysis probes (24 gauge; 10 mm in length with ~1 mm of active membrane) were lowered into the guide cannulae and perfused with artificial cerebrospinal fluid (146 nM NaCl, 1.2 mM CaCl_2_, 2.7 mM KCl, 1.0 mM MgCl_2_, pH = 7.4) at a rate of 2 μL/min. Each mouse underwent two distinct microdialysis sessions, conducted in opposite hemispheres, with the hemispheres counterbalanced across subjects within each genotype. During the first microdialysis session, mice first underwent glutamate no net-flux procedures, followed by a 1 h washout period, and then were perfused with high K+ solutions. One week later, mice underwent the 2nd microdialysis session, which began with dopamine no net-flux procedures, followed by a 1 h washout period and then NMDA perfusion. During both microdialysis sessions, dialysate collection began following 3 h of probe equilibration, baseline neurotransmitter levels were sampled for a 1 h period prior to the start of perfusion procedures, and all solutions were perfused in ascending order of concentration for 1 h each. Dialysate was stored at −80°C prior to high-pressure liquid chromatography (HPLC) determination of dopamine and glutamate levels (see below). Microdialysis probe localization within the mPFC was verified using standard Cresyl violet staining procedures, followed by an examination of tissue under a light microscope. Only data from mice in which the active membrane of the microdialysis probe was localized to the mPFC were included in the statistical analyses (see Figure 1).

### 4.4. HPLC

The HPLC system and procedures for the electrochemical detection of glutamate in the dialysate of mice, as well as the chromatographic analysis of the data, were identical to those previously by our group, e.g., [67,69,70]. The HPLC systems consisted of a Coularray detector, a Model 542 autosampler, and a Model 582 solvent delivery system (ESA Inc., Bedford, MA, USA), with a detection limit of 0.01 fg/sample (20 μL/sample onto column). The mobile phase consisted of 100 mM NaH_2_PO_4_, 22% methanol (*v*/*v*), 3.5% acetonitrile (*v*/*v*) pH = 6.75, and glutamate was separated using a CAPCELL PAK C18 MG column (5 cm; Shiseido Company Ltd., Tokyo Japan), eluting at 1.8 min. An ESA 5011A analytical cell with two electrodes (E1, +150 mV; E2, +550 mV) detected glutamate following precolumn derivatization with *o*-phthalaldehyde (2.7 mg/mL) using the autosampler. The glutamate content in each sample was analyzed by peak height and was compared with an external standard curve for quantification using ESA Coularray for Windows software (version 3.1).

### 4.5. Statistical Analyses

All cursory statistical analyses included Sex as a between-subjects factor. However, results failed to indicate any main Sex effects or interactions, likely owing to the relatively small sample sizes for female subjects (n’s = 2–5/genotype). Thus, the data were collapsed across the Sex factor for all final analyses. For the drug perfusion studies, K+ (1, 10, and 100 mM) or NMDA (1, 10, and 100 nM) were infused through the probe in ascending order for 1 h each and the data were analyzed using a Genotype X Concentration ANOVA, with Student Newman Keuls post-hoc tests as appropriate. Linear regression analyses were conducted on the plot of the average net flux of glutamate or dopamine at each neurotransmitter concentration versus the concentration of neurotransmitter infused through the probe and the point of no net flux (*y* = 0; estimate of basal extracellular levels of glutamate/dopamine), as well as the slope of regression lines (estimate of neurotransmitter clearance), were determined and analyzed using a univariate analysis of variance (ANOVA) across the Genotype factor, followed by Student Newman Keuls post-hoc tests when appropriate.

## Data Availability

The data presented in this study are available upon request from the corresponding author.
